# Microbiome of the Queensland Fruit Fly through Metamorphosis

**DOI:** 10.3390/microorganisms8060795

**Published:** 2020-05-26

**Authors:** Rajib Majumder, Brodie Sutcliffe, Phillip W. Taylor, Toni A. Chapman

**Affiliations:** 1 Applied BioSciences, Macquarie University, North Ryde, NSW 2109, Australia; phil.taylor@mq.edu.au (P.W.T.); toni.chapman@dpi.nsw.gov.au (T.A.C.); 2 Department of Environmental Sciences, Macquarie University, North Ryde, NSW 2109, Australia; brodie.sutcliffe@mq.edu.au; 3 Biosecurity and Food Safety, NSW Department of Primary Industries, Elizabeth Macarthur Agricultural Institute (EMAI), Menangle, NSW 2568, Australia

**Keywords:** gut bacteria and fungi, yeast and yeast like, Next-Generation Sequencing

## Abstract

*Bactrocera tryoni* (Froggatt) (Queensland fruit fly, or “Qfly”) is a highly polyphagous tephritid fruit fly and a serious economic pest in Australia. Qfly biology is intimately linked to the bacteria and fungi of its microbiome. While there are numerous studies of the microbiome in larvae and adults, the transition of the microbiome through the pupal stage remains unknown. To address this knowledge gap, we used high-throughput Next-Generation Sequencing (NGS) to examine microbial communities at each developmental stage in the Qfly life cycle, targeting the bacterial 16S rRNA and fungal ITS regions. We found that microbial communities were similar at the larval and pupal stage and were also similar between adult males and females, yet there were marked differences between the larval and adult stages. Specific bacterial and fungal taxa are present in the larvae and adults (fed hydrolyzed yeast with sugar) which is likely related to differences in nutritional biology of these life stages. We observed a significant abundance of the Acetobacteraceae at the family level, both in the larval and pupal stages. Conversely, Enterobacteriaceae was highly abundant (>80%) only in the adults. The majority of fungal taxa present in Qfly were yeasts or yeast-like fungi. In addition to elucidating changes in the microbiome through developmental stages, this study characterizes the Qfly microbiome present at the establishment of laboratory colonies as they enter the domestication process.

## 1. Introduction

From humans to insects, the microbiome plays an important role in host health and metabolism [1,2]. The symbiotic relationship between insects and their gut microflora is very complex, but it is essential to insect health [3]. Shin et al. [4] identified that the gut microbiome influences host gene expression in *Drosophila*, affecting host fitness through body development, nutritional metabolism, and stem cell activity. The interactions between hosts and gut microbial communities have been investigated in diverse insects, including beetles [5], mosquitoes [6], butterflies [7], silkworms [8], house flies [9], and red palm weevil [10]. A vast diversity of insect metabolic and behavioral functions have now been linked to the microbiome. Examples include the role of insect gut microbiota in the breakdown of ingested plant polysaccharides, the synthesis of secondary metabolites from sugar-based diets, the recycling of nutrients, inhibition of pathogen colonization, and the breakdown of xenobiotics or toxic materials (allelochemicals) used in plant defense [7,11,12,13,14]. A comprehensive understanding of an insect’s microbiome is an essential step toward understanding the insect’s nutritional biology and physiology and may also be a first step toward developing novel pest-management strategies for some pest insects [12,15].

Although insect microbiomes are often analyzed as a conglomerate, it is important to recognize that they comprise archaea, bacteria, and fungi, and so have substantial taxonomic breadth. Additionally, different microbial taxa have different relationships with host insects. For example, the bacteria *Providencia* spp. and the fungi *Metarhizium* spp. are known insect pathogens [16,17]. On the other hand, bacteria from the family Acetobacteraceae (includes Acetic acid bacteria) have a mutualistic relationship with the honey bee *Apis mellifera* (Hymenoptera: Apidae) [18,19] and pink sugar cane mealybug *Saccharococcus sacchari* (Cockerell) (Homoptera: Pseudococcidae) [20], both of which have a sugar-based diet. Additionally, gut-associated fungi (mostly yeast, e.g., Saccharomycetes) are an essential source of amino acids, vitamins, and enzymes, with these playing a role in detoxification, metabolism, and pheromone-production pathways [21,22,23,24,25]. While holistic studies provide a useful framework for understanding the overall role of insect microbiomes, consideration of the specific taxonomy of microbiome members is important for a detailed understanding of specific relationships and their functions.

In addition to this taxonomic and functional complexity in microbiome communities, biologically relevant temporal changes also occur in these communities. Perhaps the most dramatic example of this is the change in microbial communities through metamorphosis. Metamorphosis is a conspicuous and abrupt transformation process in which an insect undergoes a complex remodeling of its external and internal morphology [26,27,28]. All holometabolous insects undergo a metamorphosis process, starting with a larval stage, followed by a pupal stage, and finally an adult stage. Numerous holometabolous insects are economically important, due to their roles as a source of food production (e.g., honey bee), as agricultural pests (e.g., many tephritid flies) [29,30,31], as vectors of infectious diseases (e.g., sandfly and mosquito), and as important experimental models (e.g., *Drosophila melanogaster*) [26,28]. Changes in the gut microbiota during metamorphosis (e.g., butterflies [7], silkworms [8], ground-dwelling beetles [5], and longhorn beetles [32]) are most likely due to the anatomical transformations of hosts during this period [27]. In tephritid fruit flies, changes in the gut microbiota through the developmental stages have been investigated in various species of *Bactrocera*, *Ceratitis*, *Anastrepha*, and *Zeugodacus,* including *B. carambolae* [31], *B. dorsalis* [33], *B. latifrons* [34], *B. minax* (Andongma et al., 2019), *C. capitata* (aka, Medfly) [35], *A. ludens*, *A. obliqua*, *A. serpentina*, *A. striata* [29], and *Z. tau* [36], but to date there is only fragmented knowledge of how the microbiome changes through the developmental stages in Queensland fruit fly, *B. tryoni* (Froggatt) (“Qfly”).

The Qfly is highly polyphagous and is the most economically damaging insect pest of Australian horticulture [37,38,39,40]. Due to its economic importance, numerous studies have been conducted on Qfly ecology [37,41], domestication [42,43,44], production quality traits [45,46], behavior (e.g., mating performance) [47,48,49,50], larval and adult nutritional requirement [51,52,53], and microbiome [54,55,56,57,58]. Bacteria associated with wild and domesticated larvae [54,55], pupae [59], and adult Qfly [56,57,58] have been described in separate studies. Furthermore, yeasts associated with domesticated larvae [60] and adults [61] have been described. Some of these studies have used culture-dependent approaches to profile the microbiota [54,57,59], while others have used high-throughput sequencing technologies to circumvent the well-documented biases of culture-based methods [54,55,56,58]. However, there has been no comprehensive study of changes in the wild-type Qfly microbiome through metamorphosis from larvae, to pupae, to adults. Such a detailed study is important to overcome the highly constrained interpretation of comparisons across studies of different life stages, undertaken in different laboratories, and applying different techniques.

In the present study, we applied high-throughput NGS technology to profile the bacterial 16S ribosomal rRNA gene and fungal internal transcribed spacer region (ITS) obtained from larvae, pupae, and the gut of the adult Qfly. Here we addressed the microbial communities (both bacteria and fungi) present in Qflies sourced as larvae in infested fruit (generation 0/G0) that provide the starting point for laboratory rearing of Qfly from wild populations. To test the hypothesis that microbiota diversity and community structure changes through metamorphosis, we identified the dominant bacteria and fungi present at each Qfly life stage. We predicted that there would be some common dominant bacterial and fungal taxa that represented the core microbiome of Qfly across the three developmental stages, while other taxa would be specific to a developmental stage. 

## 2. Results

### 2.1. Identification of Qfly

We confirmed that all larval, pupal and adult fly samples included in this study were Qfly by both morphological and molecular testing. The genetic testing was performed by analyzing the mitochondrial cytochrome oxidase I (COI) gene using Sanger sequencing and this confirmed all 24 tested samples as Qfly. Approximately 600 additional adult Qfly in the established colony were also confirmed as Qfly. No other fly species were identified from the experimental samples.

### 2.2. Gut Bacterial Alpha and Beta Diversity During Metamorphosis

**Figure 1:**
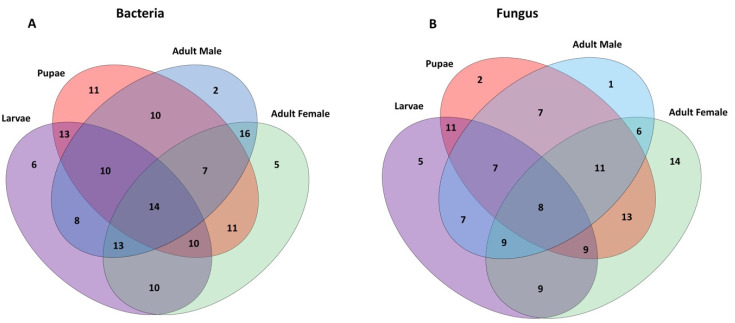
Venn diagram of the (**A**) bacterial and (**B**) fungal genera present in different developmental stages of the Qfly.

After quality filtering and the removal of chimeric sequences, and then rarefaction to 14,000 reads per sample, 74 bacterial OTUs (operational taxonomic units) were detected (Supplementary Data S1). These taxa spanned 6 phyla, 14 classes, 38 families, and 49 genera; however, despite this broad taxonomic range, only 11 genera were represented by an abundance ≥ 0.1% (Table 1). A Venn diagram analysis revealed that a subset of 14 bacterial taxa were common across all developmental stages of the Qfly (Figure 1A).

**Figure 2:**
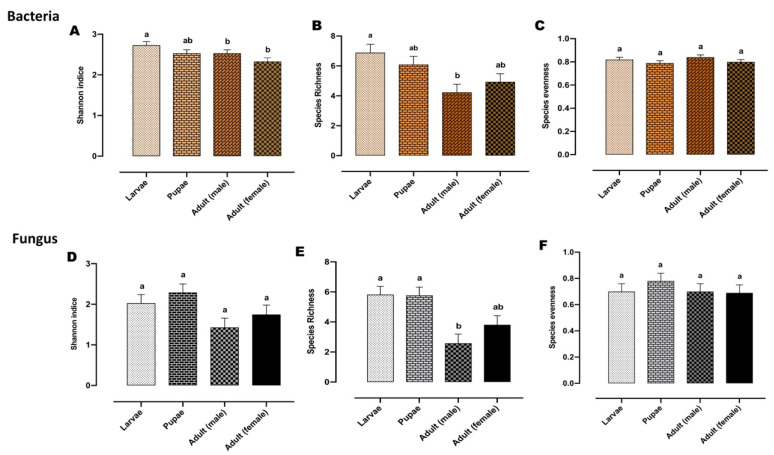
Alpha diversity of the bacterial and fungal microbiome of the Qfly developmental stages includes (**A**) Shannon indices, (**B**) species richness, and (**C**) species evenness of the bacterial microbiome. (**D**) Shannon indices, (**E**) species richness, and (**F**) species evenness of the fungal microbiome. Different letters indicate significant Tukey’s post hoc comparisons (*p* < 0.05).

Bacterial alpha biodiversity metrics, including Shannon’s biodiversity, species richness, and species evenness indices were compared between the developmental stages (Figure 2A–C). Shannon indices were significantly different between larvae and adults (both male and female flies) (*p* < 0.05) (Figure 2A). This appears to be driven by a higher species richness in the larvae, when compared with adults, although this was only significant for the male adults (Figure 2B). None of the bacterial alpha diversity metrics showed significant differences between adult males and females, or larvae and pupae.

**Figure 3:**
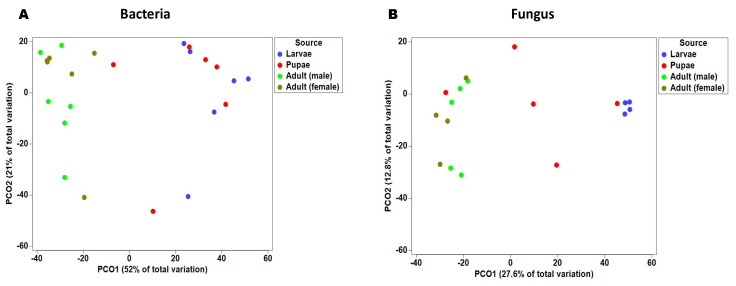
Principal co-ordinate analysis of the Qfly in the different developmental stages: (**A**) bacterial communities; (**B**) fungal communities. Different colors indicate the microbial communities in the different life stages of the Qfly.

Beta diversity of the bacterial communities at each Qfly stage was assessed by PERMANOVA analysis (pair-wise test with 999 permutation), based on Bray–Curtis similarities (Supplementary Data S1). Additionally, a principal coordinate analysis (PCoA) of this Bray–Curtis similarity matrix was used to visualize variation among host microbial communities (Figure 3A). Principal coordinate analysis (PCoA) and PERMANOVA both demonstrate a clear structural difference between bacterial communities of Qfly larvae, compared with both male and female adults (PERMANOVA < 0.05, Figure 3A; Supplementary Data S1). We found no differences between the bacterial communities of the larval and pupal microbiome (PERMANOVA test, *p* = 0.578), or between adult male and female gut microbiomes (PERMANOVA test, adult male and female *p* = 0.472, Figure 3A).

### 2.3. Bacterial Communities Associated with Metamorphosis

The relative abundance of the bacterial community members in the larval and pupal microbiome, and in adult males and females, was analyzed. At the phylum level, the most abundant taxa in the larval microbiome was Proteobacteria (98.20%), followed by Bacteroidetes (1.70%) and Actinobacteria (0.01%). In adult microbiome, Proteobacteria were observed 99.95% in females and 100% in males. Adult female flies were the only samples to host Firmicutes (0.04%), while the Actinobacteria was only found in the larvae and pupal microbiome. At the family level, the most prevalent taxa were the Enterobacteriaceae, which represented an average relative abundance of 76.1% at all developmental stages. However, they were of greatest relative abundance in adults (males 99.78% and females 98.80%), compared with the larvae (49.59%) and pupae (56.22%). Conversely, the Acetobacteraceae was observed to be highly abundant both in larvae (48.58%) and pupae (42.98%) but were substantially less abundant in adult males (0.18%) and females (1.09%). The Xanthomonadaceae was only observed in the larvae (0.03%) and pupae (0.33%). 

**Figure 4:**
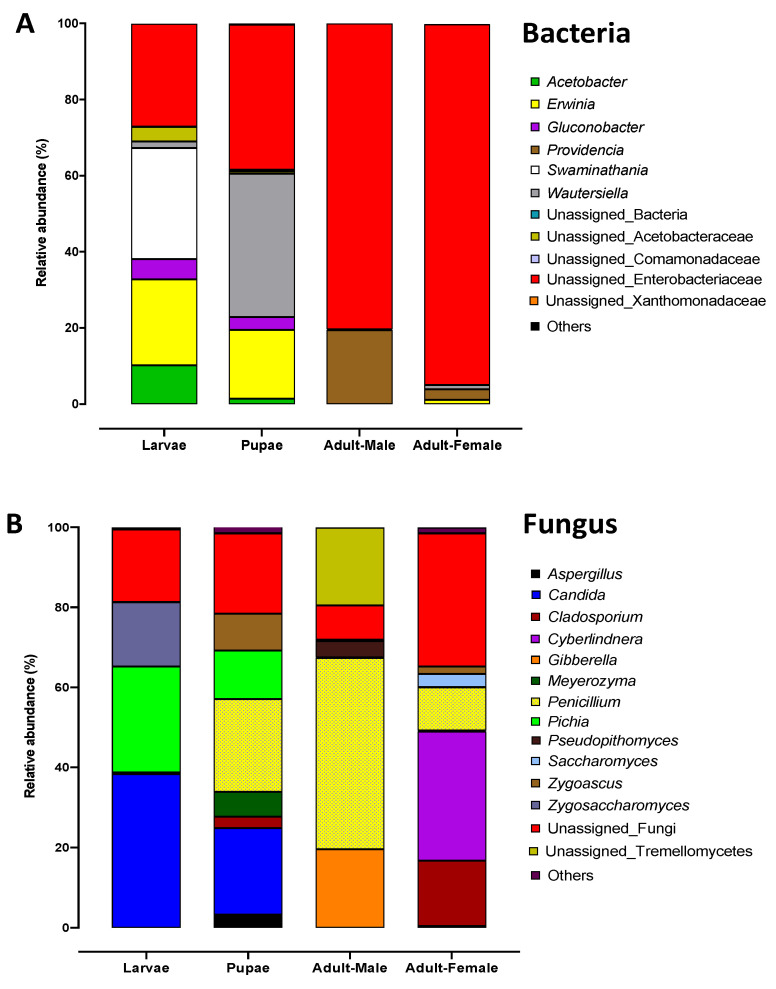
Relative abundance of the (**A**) bacterial and (**B**) fungal genus present in the different developmental stages of the Qfly.

At the genus level, bacterial taxa with the greatest relative abundance across the dataset were unassigned Enterobacteriaceae (60.1%), *Swaminathania/Asaia* (17%), *Erwinia* (10.5%), *Providencia* (5.6%), *Acetobacter* (2.9%), *Gluconobacter* (2.2%), and unassigned Acetobacteraceae (1.1%) (Figure 4A and Table 1). Highly abundant sequences of unassigned Enterobacteriaceae were reconfirmed with Geneious R10.2.3 [55], a bioinformatic software platform for sequence data analysis, using the NCBI database, and was 99.9% matched with the bacterial genus *Enterobacter*. The abundant taxa were identified across this dataset; however, these were not equally distributed among the Qfly life stages. For example, *Swaminathania/Asaia* was highly abundant both in larvae (29.2%) and pupae (37.6%) but had relatively low abundance in adults (male 0.2% and female 1.1%). Furthermore, *Erwinia* was also abundant in the larvae and pupae (22.6% and 18.1%, respectively), but represented only 1.1% in adult females, and was not detected in adult males (Table 1). In contrast, the average relative abundance of the unassigned Enterobacteriaceae (*Enterobacter*) was particularly high in adults (males 80.4% and females 94.8%, respectively), compared with larvae 27% and pupae 38.1% (Figure 4A)

### 2.4. Fungal Alpha and Beta Diversity during Metamorphosis

The fungal microbiome of 24 Qfly samples were sequenced, of which 22 were retained after quality control, and rarefaction at 1000 reads per sample. In total, 96 fungal ITS OTU sequences were identified, with taxa spanning 4 phyla, 11 classes, 35 families, and 40 fungal genera (Supplementary Data S1). Among them, only 14 fungal genera (~10%) were listed as abundant, i.e., comprising ≥ 1% of the fungal microbiome in one or more samples (Table 2). A subset of eight core fungal taxa were commonly found across all developmental stages of the Qfly, using Venn diagram analysis (Figure 1b).

Shannon’s biodiversity, species richness, and species evenness indices were illustrated in the alpha diversity of the fungal communities (Figure 2D–F). When comparing alpha diversity metrics between different developmental stages, fungal community species richness was found to be greater in larvae and pupae than in adults. This was significant for adult males, but not for adult females (*p* < 0.05) (Figure 2E). Shannon’s biodiversity index also tended to be greater in the larvae and pupae than in adults, although these comparisons were not found to be significant. 

Beta diversity of these fungal communities was explored by using a combination of principal coordinate analysis (PCoA) and PERMANOVA (pair-wise test with 999 permutation) (Figure 3B; Supplementary Data S1). Principal coordinate analysis (PCoA) axis PCO1 accounted for 27.6% of total variation seen in fungal communities and correlated with a separation of larvae and pupae samples from adult male and female samples. However, the pupae proved to be extremely disparate, with one sample clustering with the larvae, another sample clustering with the adults, and the remaining samples sitting between the two clusters (Figure 3B). Results from PERMANOVA support this separation, with larvae fungal communities significantly distinct from adult males and females (*p* < 0.05).

### 2.5. Fungal Communities Associated with the Qfly Metamorphosis

The most abundant fungal phylum was Ascomycota (94.28%), followed by Basidiomycota (4.94%). The phylum Basidiomycota was observed in the adult male gut but was rarely observed in other developmental stages. Trichocomaceae was the dominant fungal family found with an average relative abundance of 21.40%. Other abundant families included Ascomycota incertae sedis (23.16%), Pichiaceae (9.71%), Nectriaceae (4.92%), Saccharomycetaceae (4.86%), Cladosporiaceae (4.79%), Trichomonascaceae (2.83%), Debaryomycetaceae (1.57%), and Didymosphaeriaceae (1.03%). Trichocomaceae was of greater relative abundance in adult males (47.77%) compared to females and other developmental stages (female adult 10.83%, pupae 26.50%, and larvae 0.48%). The Pichiaceae was highly abundant in larvae (26.47%) and pupae (12.07%), but not in adults. Cladosporiaceae and Debaryomycetaceae were both only abundant in adult females and pupae. The Trichomonascaceae had the lowest relative abundance in larvae (0.48%), compared with other stages (male adult 47.77% female adult 10.83%, pupae 26.50%).

At the genus level, Penicillium, Candida, Pichia, Cyberlindnera, Gibberella, unassigned Tremellomycetes, Cladosporium, Zygosaccharomyces, Zygoascus, Meyerozyma, and Pseudopithomyces were found to be the most abundant fungi across the dataset (Figure 4B; Table 2). Adult males (47.8%) harbored a much higher proportion of the fungal genus Penicillium compared to adult females (10.8%) and larvae (0.4%). Further, unassigned Tremellomycetes (19.4%), Gibberella (19.6%), and Pseudopithomyces (4.1%) were abundant only in the adult male gut microbiome (Table 2). Candida and Pichia were abundant predominantly in the larval and pupal microbiome, but not in adults. Cyberlindnera (32.3%) was abundant only in the female gut and was completely absent in males and other developmental stages. Conversely, Qfly larvae contained Zygosaccharomyces at a relative abundance of 16.1%, but these were not found in other life stages.

## 3. Discussion

The present study identifies and characterizes the microbial communities present in the different developmental stages of wild-type Qfly (G0) at the point of entry into laboratory rearing. The use of high-throughput sequencing methods to profile both bacterial and fungal elements of the microbiome circumvents the well-known difficulties in isolating microbes through traditional, culture-dependent methods. Indeed, a number of the taxa identified in both bacterial and fungal datasets were novel, having no closely related described culture representatives. This approach enabled us to assess biodiversity independent of culturing, and to examine how the Qfly microbiota changes through development and between the adult sexes, without the biases inherent to culture-based approaches.

For both bacterial and fungal microbiota communities, substantial and significant shifts in beta diversity occurred between larvae and adults. This has been observed in other tephritid fruit fly species of the genera *Bactrocera*, *Zeugodacus*, *Ceratitis*, and *Anastrepha* [29,30,31,33,36,62], as well as in other insects, including butterflies, beetles, and mosquitoes [5,6,7,32]. Interestingly, the pupae appear to be a transitionary phase for the microbial communities. Minor shifts occurred in the microbial communities of pupae to make them less like larvae but not the same as adults. For bacterial communities, these shifts resulted in a significant decline in overall diversity for adult males and females, compared with the larvae. During the pupal period, the gut microbiome undergoes minimal metabolic activity [15]. Like other holometabolous insects (e.g., bark beetle *Dendroctonus rhizophagus*), morphological changes during Qfly metamorphosis might impact the bacterial community structure [63]. Previous research on Medfly and butterfly gut bacteria during metamorphosis is consistent with our findings [35,64]. Similarly, Moll et al. [65] found that the gut community structure of the mosquitoes (Diptera: Culicidae) *Anopheles punctipennis* (Say), *Culex pipiens* (L.), and *Aedes aegypti* (L.) changes rapidly during metamorphosis. The environment, diet, and developmental time can all be key factors affecting gut microbial diversity in insects [15,66,67] and might each contribute to the results of the present study. Our results are also consistent with previous findings in microbial analysis across developmental stages of *B. dorsalis* [68,69], *B. carambola* [30], *B. minax* [62], *C. capitata* [35], four *Anastrepha* fruit flies—*A. ludens*, *A. obliqua*, *A. serpentina*, and *A. striata* [29]—and *Z. tau* [36].

This study revealed that the bacterial community was dominated by proteobacteria comprising 99.52% of identified taxa found across Qfly larvae, pupae, and adults (both male and female). This trend has also been observed in other *Bactrocera* species, including in studies of bacteria present at various stages of *B. carambola* metamorphosis [30]. Furthermore, these findings are consistent with the high abundance of Proteobacteria reported in adults of other tephritid species, including *B. cacuminata*, *B. dorsalis*, *B. jarvisi*, *B. minax*, *B. neohumeralis*, *A. ludens*, *A. obliqua*, *A. serpentina*, *A. striata*, *Z. tau*, and *C. capitata* [29,31,33,35,36,56,62,70]. Within the Proteobacteria, different families were associated with different life stages. For example, Enterobacteriaceae was found to be the most dominant family in adult Qfly, but Acetobacteraceae were abundant in both larval and pupal stages; Reference [58] also found Enterobacteriaceae to be dominant in the gut microbiome of wild Qfly adults. Enterobacteriaceae are mostly transmitted to Qfly larvae via vertical transmission during oviposition [54,55]. In *B. oleae* larvae, these bacteria fix nitrogen and perform pectinolysis in the gut [71,72]. Additionally, previous studies of *C. capitata* larvae showed that supplementation of *Enterobacter* spp. in the diet decreases developmental time, and increases pupal weight and mating performance during mass rearing [73,74,75,76]. Within the family Enterobacteriaceae, we identified the bacterium *Erwinia* in the larvae, pupae, and adult female Qfly microbiome. *Erwinia* plays role in nitrogen fixation and helps insects to sustain themselves in environments with limited oxygen [77]. It is likely that these functions are required at all life stages in the Qfly, potentially explaining the consistency in their relative abundances across the dataset. In comparison, the bacterium *Providencia* was only found in adult male and female gut microbiota.

We also observed the dominance of three Acetobacteraceae bacterial genera, *Swaminathania/Asaia*, *Acetobacter*, and *Gluconobacter*, in the immature stages (larvae and pupae) of the Qfly and at very low abundance in adults. A similar observation was reported for *B. dorsalis* [33]. Previously, *Swaminathania/Asaia* was also detected at low abundance in adults of both Qfly [56,58] and *B. oleae* [78]. Although the role of the *Swaminathania/Asaia* is still unknown in tephritid fruit flies [54,58], these bacterial taxa have also been reported in the adult *D. melanogaster*, *Anopheles*, and *Aedes* mosquitoes, as well as the honeybee *Apis mellifera* [79]. In addition, Chouaia et al. [80] observed that lack of *Swaminathania/Asaia* spp. delayed larval development in *Anopheles stephensi*. *Acetobacter pomorum* and *Swaminathania/Asaia* supply essential nutrients that improve larval development in *Drosophila* and *Anopheles gambiae* mosquitoes [4,81]. Therefore, we hypothesize the reason behind life stage-specific variation in abundance is that *Swaminathania/Asaia* might be an essential symbiont during the larval stages to maintain larval development but is much less important in adult stages and found in low abundance. Furthermore, it might be that the larval gut bacterial communities (mostly *Swaminathania*, *Acetobacter*, and *Gluconobacter*) are favored by the fruit-based carbohydrate-rich diet, whereas adults consume more protein [68]. Considering this, the bacterial communities in adults (mostly from the bacterial family Enterobacteriaceae) may be more focused on protein metabolism than the larval microbiome.

The fungal microbiome of the Qfly during development includes various types of fungi and yeast. Our study identified many fungi and yeast that are generally difficult to isolate or culture via traditional methods [82,83,84,85]. This study on the fungal microbiome of Qfly across life stages using NGS appears to be the first not only in Qfly but also in any tephritid species.

In the present study, alpha diversity and beta diversity showed similar trends for bacterial and fungal communities and further indicated a simple structure in the fungal community across stages. Fungal species richness was significantly greater in the larval stage than in adult male microbiome. Among all developmental stages of the Qfly, species richness was highest in the larvae, followed by pupae, while adult males contained the lowest number of fungal species. Yeast and yeast-like fungi produce proteins, carbohydrates, and lipids that can provide important nutrition to host insects [25,86,87]. Yeast and yeast-like fungi present in the insect gut also contribute to amino acid and fatty acid metabolic pathways, and, consequently, development through metamorphosis can be compromised if yeasts are not present [25,88]. Although Qflies do not feed during the pupal stage, they maintain a microbiome that might support metabolic activities during this stage. Based on PCoA ordination plots, the fungal microbial communities varied across the Qfly developmental stages, with larval and pupal stages being clearly separated from the adults, but retaining a similarity to each other. It might be that the morphological transformation of the Qfly from the larval stage to pupal stage causes a reduction of metabolic activities, which impacts on the fungal communities that transmit to the adult stage [63,89]. The structure of the gut microbial communities of insects can be modified through enzyme production according to host morphology during metamorphosis and diet [90]. Our findings are somewhat similar to those of Hu et al. [91] for fungal community structure of the Chinese white pine beetle *Dendroctonus armandi* across different developmental stages. Overall, both bacterial and fungal diversity followed a trend of decreasing from the larval stage to the adult stage. Additionally, the PCoA plots also showed similar results with the bacterial and fungal communities of the larvae and pupae clustering together and separating from the adults, while the adult male and female gut microbial communities clustered closely together.

At the phylum level, Ascomycota was present at the highest abundance in every developmental stage of the Qfly microbiome. The greatest number of ascomycetes were associated genera of *Penicillium*, *Candida*, *Pichia*, *Cyberlindnera*, *Gibberella*, *Cladosporium*, *Zygosaccharomyces*, *Zygoascus*, *Meyerozyma*, *Aspergillus*, and *Saccharomyces* (Figure 4B). These fungi are mostly identified as single-cell fungi commonly known as the budding yeasts [7]. Different types of yeast were found at different development stages of Qfly. Therefore, it might be that the Qfly ingests yeast as a food source during larval and adult stages and few strains are able to transmit across all developmental stages. The fungal taxa *Candida* and *Pichia* were highly abundant in the larval and pupal stage but were comparatively rare in adults. Similarly, a comprehensive fungal analysis of adult wild *B. oleae* did not identify any fungi associated with *Candida* and *Pichia* [84]. In contrast, these fungi have been reported in other insects including spotted wing *drosophila*, *Drosophila suzukii* (Matsumura) (Diptera: Drosophilidae) [92], and *Agrilus mali* (Coleoptera: Buprestidae) [89]. This result indicated that certain fungi present in the larval and pupal stages did not transmit to the adults. Similarly, the yeast genus *Saccharomyces* was only found in gut of adult female Qfly. Furthermore, *Saccharomyces* have been detected in the adult stage of *B. oleae* (Diptera: Tephritidae) [93]. Vega and Blackwell [25] demonstrated that *Saccharomyces* and *Candida* produce digestive enzymes, including *β*-glucosidases, xylases, and cellulases, to help digestion in the host insect. These yeasts can play a vital role on detoxification of toxic compounds from plants [67]. 

The percentage of the relative abundance of *Penicillium* was observed to be significantly lower in the larval stage and increased from the pupal microbiome to adults. Previously, *Penicillium* has been detected in other fruit flies, including *C. capitata* [94] and *B. oleae* pupae and adults (both male and females) [84]. Deutscher et al. [60] isolated *Penicillium* from the midgut of Qfly larvae, using culture-based methods. Various toxigenic species are included under the fungal genus *Penicillium*, and most produce mycotoxin. *Penicillium chrysogenum* and *P. notatum* are used to produce the commercial antibiotic Penicillin [95,96]. However, Konstantopoulou and Mazomenos [94] demonstrated that toxin from *Penicillium* was not toxic to insects. A possible explanation is that *Penicillium* might be capable of outcompeting other fungi in certain conditions or environments, for example, in the low pH levels of the Qfly gut. 

Furthermore, we observed fungal genera including *Cladosporium*, *Zygoascus*, and *Meyerozyma* present in adults, mostly in female gut fungal communities. The genus *Cladosporium* has also been found to be abundant in the gut of both male and female *B. oleae* [84]. *Cladosporium* associated with sooty mold communities are mainly abundant in plant phylloplane and carpoplane [97,98]. Additionally, Bensch et al. [99] demonstrated that the fungal genus *Cladosporium* can cause plant diseases. *Cladosporium* was found at low levels in the pupal stage of Qfly but became highly abundant in adult females. It might be possible that *Cladosporium* presents at very low abundance in later larval stages and then only becomes detectable at the pupal stage of the Qfly [100]. The opposite was found in the genus *Zygoascus*. This yeast was highly abundant in the Qfly pupal stage but was much less abundant in adults. Moreover, the yeast genus *Zygoascus* has been reported in beetles [101]. The fungal genera *Meyerozyma* and *Aspergillus* were abundant in Qfly pupae. Additionally, *Meyerozyma* have been found in burying beetles *Nicrophorus vespilloides* [102] and in the hindgut of carrion beetles (Coleoptera, Silphidae) [103]. *Aspergillus* has also been detected in *B. oleae* [84]. We found that some plant pathogens were associated with some Qfly developmental stages. We hypothesized that the Qfly might act as a host carrier of these fungal pathogens and ingest them with food or from the environment. For example, fungal species of *Colletotrichum* are sourced by *B. oleae* from olive fruits and are present for at least a part of their life cycle with this pest fruit fly. *Colletotrichum* causes olive anthracnose that greatly effects the quality of both fruits and oil [104,105]. *Bactrocera oleae* might be an important disease carrier that spreads *Colletotrichum* among olive fruits [84,106]. A group of unidentified fungi (mostly yeasts) were found in every developmental stage of Qfly but were highly abundant in the larvae. These fungi are likely transmitted horizontally to the larvae from infested fruits during ingestion of fruit flesh, and many of these may be transient.

## 4. Materials and Methods

### 4.1. Qfly Sample Collection

Infested pomegranates *Punica granatum*, green apples *Malus pumila*, and quinces *Cydonia oblonga* were collected from different geographic locations in New South Wales (NSW) and Victoria (VIC), Australia (Table 3). The infested fruits were collected from under trees, and most were over-ripe. After collection, all fruits were stored in 60 L plastic bins (Award, Bunnings Warehouse, Greenacre, NSW, Australia) that contained a layer of fine vermiculite (1.0 cm depth) (Grade 1, Sage Horticultural, Hallam, VIC, Australia). All containers with infested fruits were placed in a controlled-environment laboratory (25 ± 0.20 °C, 65 ± 3% RH and 11 h : 1 h : 11 h : 1 h light : dusk : dark : dawn photoperiod). Collected pupae were transferred in mesh cages (Megaview Bugdorm 44545, 47.5 × 47.5 × 47.5 cm, MegaView Science Co., Ltd., Talchung, Taiwan). After emergence, the emerged flies were also placed in the same mesh cages. The adult flies were supplied with hydrolyzed yeast (MP Biomedicals, Irvine, CA, USA, Cat. No. 02103304) and commercial sucrose (CSR® White Sugar, Maribyrnong VIC, Australia). This Qfly colony of Generation 0 (G0) was considered as wild-type, whereas larvae and pupae were collected from the natural host fruits. The adults, however, were fed hydrolyzed yeast with sugar (2:1) and water for 15 days. Each developmental stage of the Qfly from G0, 3^rd^ instar larvae (*n* = 6), 8 days old pupae (*n* = 6), and 15 days old sexually mature adults, both male (*n* = 6) and female flies (*n* = 6), were collected for high-throughput NGS.

### 4.2. Sample Preparation

For sample processing, Qfly larvae, pupae, and adult flies (male and female separately) were surface sterilized with 0.5% Tween 80 (Sigma-Aldrich, St. Louis, MO, USA, Cat. No. 9005656), 0.5% Bleach (Sodium hypochlorite) (Sigma-Aldrich, St. Louis, MO, USA, Cat. No. 7681529), and 80% Ethanol (Sigma-Aldrich, St. Louis, MO, USA, Cat. No. 65175) for 30 s, and rinsed 3 times in 1M sterile phosphate-buffered saline (1× PBS) for 30 s [55]. The PBS from the 2^nd^ and 3^rd^ washes were kept and 100 µL spread-plated onto five types of microbial growth media (de Man, Rogosa and Sharpe Agar, Tryptone Soya Agar, Macconkey Agar, Potato Dextrose Agar and Yeast-dextrose Agar medium) (Sigma-Aldrich, St. Louis, MO, USA), to confirm the surface sterilization of the insects. All plates were incubated at 35 °C for 24 to 48 h post-sterilization, and the guts of adult flies were dissected under a stereomicroscope (Leica MZ6, Leica®, Wetzlar, Germany) [55]. Surface sterilization of the larvae, pupae, and adult flies (before dissection) was found to be effective as there was no microbial growth detected in any growth medium after 24 to 48 h incubation. Using sterile pestles, larvae, pupae, and dissected guts from the adults were homogenized separately in a sterile solution of Brain Heart Infusion (BHI) broth (Oxoid Ltd, Basingstoke, UK, Lot # 1656503) and 20% Glycerol (Sigma-Aldrich, St. Louis, MO, USA, Lot # SHBG2711V). Each sample was split into two separate cryovial tubes (Simport Scientific, Saint-Mathieu-de-Beloeil, QC, Canada) for both cytochrome oxidase I (COI) gene identification and high-throughput NGS analysis. All samples were preserved at −80 °C for future use. All procedures were completed in a sterile environment (Biological Air Clean Bench, safe 2020 1.2, Thermo Scientific, Dreieich, Germany).

### 4.3. Qfly Identification Using Mitochondrial Cytochrome Oxidase I (COI) Gene

The mitochondrial cytochrome oxidase I (COI) gene sequencing of all samples for Qfly identification was performed according to the method described in Reference [55]. In brief, Isolate II genomic DNA (Bioline, Memphis, TN, USA, Cat. no. BIO-52065) was used to extract DNA from Qfly samples, following the manufacturer’s protocol. The Invitrogen™ Qubit® dsDNA High Sensitivity (HS) Assay Kit (Life Technologies, Eugene, OR, USA) was used to determine the DNA extract concentrations. Standard LCO1490 ⁄ HCO2198 primers were used to amplify a 700 bp segment of the CO1 gene [55,107].

All PCR amplifications were performed in an Eppendorf thermocycler (Eppendorf, Hamburg, Germany), using a MyTaqTM HS Mix (Bioline, Memphis, TN, USA), 400 nM of the primers LCO1490 and HC02198 [55], and thermocycling conditions of 95 °C for 2 min, followed by 35 cycles of 95 °C for 15 s, 50 °C for 30 s, and 72 °C for 45 s, and 5 min at 72 °C. Amplicons were visualized, using electrophoresis on a 1% agarose gel (110v for 45 min). Purified amplicons were sequenced, using the LCO1490 primer by the Australian Genome Research Facility (AGRF) (The Westmead Institute, 176 Hawkesbury Rd, Westmead, NSW 2145, Australia). The sequences were trimmed, using Geneious v. 9.1.14 (https://www.geneious.com/) [108] and compared against the National Center for Biotechnology Information (NCBI) nonredundant nucleotide collection, using the Mega-BLAST algorithm [109] available on NCBI’s BLAST web interface [110]. In addition to this molecular confirmation, microscopic examination of larval morphological features was carried out prior to DNA extraction [111]. Additional confirmation was gained through morphological observation of emerged adult flies under a stereomicroscope (Leica MZ6, Wetzlar, Germany) [55].

### 4.4. Qfly Microbiome Profiling

DNA was extracted with a DNeasy Power Lyzer Power Soil Kit-100 (Qiagen, Hilden, Germany) (Cat. no. 12888-100) for each sample, following the manufacturer’s procedure. DNA extracts were then quantified by an Invitrogen™ Qubit® dsDNA High Sensitivity (HS) Assay Kit (Life Technologies, Eugene, OR, USA). the Australian Genome Research Facility (University of Adelaide, Plant Genomics Centre, Hartley Grove, Urrbrae, SA 5064, Australia) performed the PCR amplification and sequencing. For bacterial identification, the V1-V3 16S rRNA region was amplified by using primers 27F (5′AGAGTTTGATCMTGGCTCAG-3′) and 519R (3′ GWATTACCGCGGCKGCTG-5′) [55]. For the fungi, the ITS region of RNA operon was amplified by using the fungal-specific forward primer ITS1F (5’-CTTGGTCATTTAGAGGAAGTAA-3’) and the reverse primer ITS2 (5’-GCTGCGTTCTTCATCGATGC-3’) [112,113,114]. Reactions contained 1X AmpliTaq Gold 360 mastermix (Life Technologies, Eugene, OR, USA) and 0.20 µM of each forward and reverse primer with 25 µL of DNA. The cycling conditions of the PCR consisted of denaturation at 95 °C for 7 min, 35 cycles of 94 °C for 45 s, 50 °C for 60 s, and 72 °C for 60 s, as well as a final extension of 72 °C for 7 min. A secondary PCR was used to adhere sequencing adaptors and indexes to the amplicons. Primerstar max DNA Polymerase was used for secondary PCR amplicon generation from Takara Bio Inc., Shiga, Japan (Cat. No. #R045Q). The resulting amplicons were measured by fluorimeter (Invitrogen Picogreen, Thermo Fisher Scientific, NSW, Australia) and normalized [115]. The equimolar amounts of each sample were pooled and quantified by qPCR prior to sequencing (Kapa qPCR Library Quantification kit, Roche, Basel, Switzerland). The resulting amplicon library was then sequenced on the Illumina MiSeq platform (San Diego, CA, USA), with 2 × 300 base pairs paired-end chemistry [116].

### 4.5. Sequence Data Processing

Both bacterial 16s rRNA and fungal ITS amplicons were processed by first aligning forward and reverse reads, using PEAR (v.0.9.5) [117]. For the 16S rRNA data, quality filtering, clustering, and taxonomic assignments were achieved by using the USEARCH tools [55,118,119] and rdp_gold database as a reference (Ribosomal database project, https://rdp.cme.msu.edu) [55,120]. Any 16S rRNA OTUs with taxonomic assignments to eukaryotic organelles (e.g., chloroplast) were removed from the dataset. The ITS sequence data processing was completed, and sequences were quality filtered, using USEARCH tools. Full-length duplicate sequences were removed and sorted by abundance. Singletons or unique reads in the dataset were discarded. Sequences were clustered followed by chimera filtering, using the Unite database as reference. Reads were mapped back to OTUs with a minimum identity of 97%, to obtain the number of reads in each OTU. QIIME taxonomy was assigned by using the Unite database [121] (Unite v.7.1 Dated: 22.08.2016). An in-house python script was applied for rarefaction. To maintain equal sequence depth among all samples, we then rarefied to 14,000 reads per sample for bacteria and 1000 reads for fungi, repeating these 50 times and averaging the counts, to obtain a representative rarefaction. Samples with < 14,000 reads and < 1000 reads for bacteria and fungi, respectively, were removed from analysis. The data were normalized as the percentage of relative abundance, which is henceforth referred to as the OTU table (Supplementary Data S1) [55]. All the figures of bacterial relative abundance at genus levels were plotted in Prism 8, v.8.0.1(145), GraphPad software, Inc., San Diego, CA, USA).

### 4.6. Statistical Analysis

The bacterial and fungal OTU tables were imported into Primer-E v7 for analysis [55,114,122,123]. The OTU table contained the number of read counts for each OTU detected for each sample. In brief, all statistical testing was performed on fixed factors associated with each developmental stage of the Qfly, both for bacterial and fungal analysis (larvae, pupae, and adult male and female) from which 6 replicates were collected. The DIVERSE function was used to generate univariate biodiversity metrics, species richness, Pielou’s evenness, and Shannon’s biodiversity indices. Statistical differences between these metrics were assessed in JMP Statistical Software Version 10.0.0 (SAS Institute, Cary, NC, USA), using one-way analysis of variance (ANOVA) and Tukey–Kramer’s post hoc analysis [55]. To observe the taxonomic compositional changes in the bacterial and fungal communities, the OTU table was first log transformed, using Primer-E v7. A Bray–Curtis similarity matrix was derived from these transformed data, and a permutation analysis of variance (PERMANOVA) pairwise comparison was conducted to compare all community samples. A *p*-value of < 0.05 was considered statistically significant. Furthermore, ordination plots of these communities were visualized by using principal coordinates analysis (PCoA) in Primer-E [55].

### 4.7. Data Availability Statement

The Illumina sequences were deposited and publicly available in the NCBI GenBank, under Bio-project PRJNA556787.

## 5. Conclusions

The present study demonstrates that microbial communities both of bacteria and fungi differ between the larvae and adults of the Qfly. Our findings contribute to increased understanding of the bacteria and fungi present in the Qfly through development and their role as commensals and pathogens. In addition to illuminating the intimate life-stage-specific ecological relationships between the Qfly and its microbiota, this knowledge may also have more applied value for efforts to manage this challenging pest species. For example, this knowledge may facilitate manipulation of the microbiome to improve the artificial diet both for the larvae and adults, thereby improving the quality of artificially reared Qfly, and it may also provide a useful starting point for the development of novel pest-management solutions.

## Figures and Tables

**Table 1:** Taxonomic identification of the of the 11 most abundant bacterial taxa in the Qfly across all developmental stages (% based on genus level).

Domain	Phylum	Class	Order	Family	Genus	Larvae	Pupae	Adult Male	Adult Female
Bacteria	Proteobacteria	Gammaproteobacteria	Enterobacteriales	Enterobacteriaceae		27.0%	38.1%	80.4%	94.8%
Bacteria	Proteobacteria	Alphaproteobacteria	Rhodospirillales	Acetobacteraceae	*Swaminathania/Asaia*	29.2%	37.6%	0.2%	1.1%
Bacteria	Proteobacteria	Gammaproteobacteria	Enterobacteriales	Enterobacteriaceae	*Erwinia*	22.6%	18.1%	0.0%	1.1%
Bacteria	Proteobacteria	Gammaproteobacteria	Enterobacteriales	Enterobacteriaceae	*Providencia*	0.00%	0.00%	19.4%	2.8%
Bacteria	Proteobacteria	Alphaproteobacteria	Rhodospirillales	Acetobacteraceae	*Acetobacter*	10.2%	1.4%	0.0%	0.0%
Bacteria	Proteobacteria	Alphaproteobacteria	Rhodospirillales	Acetobacteraceae	*Gluconobacter*	5.3%	3.4%	0.0%	0.0%
Bacteria	Proteobacteria	Alphaproteobacteria	Rhodospirillales	Acetobacteraceae		3.8%	0.6%	0.0%	0.0%
Bacteria	Bacteroidetes	Flavobacteriia	Flavobacteriales	Weeksellaceae	*Wautersiella*	1.7%	0.0%	0.0%	0.0%
Bacteria	Proteobacteria	Betaproteobacteria	Burkholderiales	Comamonadaceae		0.0%	0.4%	0.0%	0.0%
Bacteria	Proteobacteria	Gammaproteobacteria	Xanthomonadales	Xanthomonadaceae		0.0%	0.3%	0.0%	0.0%
Bacteria						0.1%	0.1%	0.0%	0.0%

**Table 2:** Taxonomic identification of the 14 most abundant fungal taxa in the Qfly across all developmental stages (% based on genus level).

Domain	Phylum	Class	Order	Family	Genus	Larvae	Pupae	Adult Male	Adult Female
Fungi	Ascomycota	Eurotiomycetes	Eurotiales	Trichocomaceae	*Penicillium*	0.4%	23.2%	47.8%	10.8%
Fungi	Ascomycota	Saccharomycetes	Saccharomycetales	Incertae-sedis	*Candida*	38.3%	21.5%	0.0%	0.4%
Fungi	Ascomycota	Saccharomycetes	Saccharomycetales	Pichiaceae	*Pichia*	26.5%	12.1%	0.1%	0.1%
Fungi	Ascomycota	Saccharomycetes	Saccharomycetales	Incertae-sedis	*Cyberlindnera*	0.0%	0.0%	0.0%	32.3%
Fungi	Ascomycota	Sordariomycetes	Hypocreales	Nectriaceae	*Gibberella*	0.0%	0.0%	19.6%	0.1%
Fungi	Basidiomycota	Tremellomycetes				0.0%	0.0%	19.4%	0.0%
Fungi	Ascomycota	Dothideomycetes	Capnodiales	Cladosporiaceae	*Cladosporium*	0.0%	2.9%	0.0%	16.3%
Fungi	Ascomycota	Saccharomycetes	Saccharomycetales	Saccharomycetaceae	*Zygosaccharomyces*	16.1%	0.0%	0.0%	0.0%
Fungi	Ascomycota	Saccharomycetes	Saccharomycetales	Trichomonascaceae	*Zygoascus*	0.0%	9.2%	0.3%	1.8%
Fungi	Ascomycota	Saccharomycetes	Saccharomycetales	Debaryomycetaceae	*Meyerozyma*	0.0%	6.2%	0.0%	0.1%
Fungi	Ascomycota	Dothideomycetes	Pleosporales	Didymosphaeriaceae	*Pseudopithomyces*	0.0%	0.0%	4.1%	0.0%
Fungi	Ascomycota	Eurotiomycetes	Eurotiales	Trichocomaceae	*Aspergillus*	0.0%	3.3%	0.0%	0.0%
Fungi	Ascomycota	Saccharomycetes	Saccharomycetales	Saccharomycetaceae	*Saccharomyces*	0.0%	0.0%	0.0%	3.3%
Fungi						18.2%	20.1%	8.6%	33.3%

**Table 3:** Fruit types and origin used for the wild-type *Bactrocera tryoni* colony (G0).

Geographic Location of Collection	Fruit Source and Number of Fruits Collected	Collection Date
Coomealla, NSW GPS: Lat 34° 5¡ä50.97", Long 142° 3¡ä7.21"	Pomegranate 37 pieces	5/05/17
St. Germains, Between Tatura and Echuca in Victoria GPS: Lat 36°10’48.86", Long 145° 8’50.74"	Green Apple 41 pieces	05/05/17
Downer road between Tatura and Toolamba in Victoria GPS: Lat 26°38’34.92", Long 152°56’22.99"	Quince 52 pieces	05/05/17

## Supplementary Materials

The following are available online at https://www.mdpi.com/2076-2607/8/6/795/s1.
